# An FAQ dataset for E-learning system used on a Japanese University

**DOI:** 10.1016/j.dib.2019.104001

**Published:** 2019-05-24

**Authors:** Yasunobu Sumikawa, Masaaki Fujiyoshi, Hisashi Hatakeyama, Masahiro Nagai

**Affiliations:** Tokyo Metropolitan University, Japan

## Abstract

In this data article, we present an FAQ dataset written in Japanese and its translation to English in order to train chatbot models for e-learning systems. We first collected raw Q&A data reported as the difficulties from April 2015 to July 2018 by users of the e-learning system introduced at Tokyo Metropolitan University. We then divided them into 11 categories according to features provided by the e-learning system. Finally, we integrated questions with the same answers in order to create the FAQ form. The dataset contains 427 questions and 79 answers that were examined by experts with experience in using the e-learning system for more than three years. Using this dataset, we performed statistical analyses to evaluate the qualities of the FAQ dataset. The proposed applications of the dataset include not only academic research but also activities; for example, translating from Japanese to another one like Chinese, adapting/modifying our dataset for another e-learning system, and developing language models to obtain highly accurate responses from chatbots.

**Table:** Specifications Table

Subject area	*Data mining, natural language processing*
More specific subject area	*Text classification for e-learning system.*
Type of data	*Database*
How data was acquired	*Log data asked by real users of our e-learning system.*
Data format	*Processed*
Experimental factors	*This dataset is created from raw Q&A logs.*
Experimental features	*Creating FAQ data were created by manual inspections of four experts. Cluster analyses were performed to evaluate the qualities of the created FAQ data.*
Data source location	*This dataset is stored in Zenodo. Its URL is**https://doi.org/10.5281/zenodo.2783642*.
Data accessibility	*Zenodo is a public repository.*
Related research article	*Yasunobu Sumikawa, Masaaki Fujiyoshi, Hisashi Hatakeyama, and Masahiro Nagai "Supporting Creation of FAQ Dataset for E-learning Chatbot", Intelligent Decision Technologies, Smart Innovation, IDT'19, Smart Innovation, Systems and Technologies, Springer, 2019.*

**Table:** 

**Value of the Data** • This dataset provides an FAQ dataset created by raw Q&A data about usage of the e-learning system introduced at Tokyo Metropolitan University. The Q&A data is collected from real users of the e-learning system. • This dataset is useful for training chatbot models as a labeled dataset because this is the first available dataset for training the models in e-learning written in Japanese. • We provide definitions of category schemes for answers to perform statistical analyses in order to measure/evaluate dataset quality for comparison with other datasets or the creation of new datasets.

## 1 Data

The published dataset (See metadata in Table 1) is organized to train chatbot models specifically for an e-learning system. The dataset contains questions asked by students or teachers and their answers in practice. In the dataset, there are Q&A data in Japanese (Answers.csv, Questions.csv, and Categories.csv) and English (Answers_english.csv, Questions_english.csv, and Categories_english.csv), categorization data for answers (Answer2Category.csv) and questions (Answer2Tag.csv).

**Table 1:** Database files.

File name	Content	Columns
Answers.csv	Texts of answers.	AID: IDs for answers.
		Text: Texts of answers in Japanese.
Questions.csv	Texts of questions.	Text: Texts of questions in Japanese.
		AID: IDs of answers corresponding to a question in the same line.
Categories.csv	Titles of categories.	CID: IDs for categories.
		Title: Titles of the categories in Japanese.
Answer2Category.csv	Pairs of answers and categories.	AID: IDs for answers.
		CID: IDs for categories.
Answer2Tag.csv	Titles of answers	AID: IDs for answers.
		Tag: Titles for answers.
Answers_english.csv	Texts of answers in English.	AID: IDs for answers
		Text: Texts of answers in English.
Questions_english.csv	Texts of questions in English.	Text: Texts of questions in English.
		AID: IDs of answers corresponding to a question in the same line.
Categories_english.csv	Titles of categories and their English names.	CID: IDs for categories.
		Title: Titles of the categories in Japanese.
		English Title: Titles of the categories in English.

The dataset also includes the results of statistical analyses, which are described in Section “**Statistical Analysis**” and Table 4, for comparing our study performed in Ref. [1] with subsequent new research on chatbots or making a new dataset without reducing the quality of the dataset specifically for another e-learning system by checking their scores.

**Table 2:** Statistics of the whole dataset published in this paper.

Number of categories	11
Average Number of answers per category	6.58
Average Number of questions per answer	30.58

**Table 3:** Numbers of the questions and answers in each category.

	C1	C2	C3	C4	C5	C6
Questions	9	13	17	3	3	11
Answers	50	68	89	15	19	66
	C7	C8	C9	C10	C11	*Total*
Questions	5	2	8	3	5	*79*
Answers	25	11	44	15	25	*427*

**Table 4:** Files including scores of statistics.

File name	Content
basic_stats_chvals.tsv	Scores of basic statistics. We used Eq. 1 in “ch val” raws.
inner_class.tsv	Scores of four measurements listed above from 2 (MI) to 5 (JS). In this file, we ignore all tags.
inner_class_inter_tag.tsv	Scores of the four measurements listed above from 2 (MI) to 5 (JS). This file calculates the scores between different tags for each category.
inner_tag.tsv	Scores of the four measurements from MI to JS. We calculate them between all combinations of data for each tag.
inter_class.tsv	Scores of the four measurements from MI to JS. This file calculates them between all combinations of different categories.
inter_class_inter_tag.tsv	Scores of the four measurements from MI to JS. In this file, we create combinations of tags from different categories.
inter_tag.tsv	This file calculates the four measurements for all combinations of tags including same and different categories.

## 2 Experimental design, materials, and methods

### 2.1 Data Collection

Raw data were collected by logs that recorded/collected the questions asked by users of our e-learning system and the answers provided by system engineers who managed the e-learning system for more than three years. The collection processes were performed from April 1, 2015 to July 31, 2018. The dataset contains a total of 200 pairs of questions and answers.

### 2.2 Creating FAQ

After collecting Q&A data, FAQ data were created by combining the raw answers that are semantically similar to each other. If the number of questions for an answer is less than five, then several new questions were added by paraphrasing using suggestion tools proposed in Ref. [1]. When the new questions were added, four experts with experience in using and managing the e-learning system for more than three years examined whether the results were correct or not.

From this FAQ data, we trained a chatbot from these Q&A data and then confirmed that the chatbot correctly predicted over 81% in terms of macro-average F_1_-score [1]. Note that this result is confirmed only from the Japanese version's dataset. To increase usability and to check the validity of the dataset, we translate the dataset to English. As the English dataset is a translation, the number of Q&A data and the organization of them are the same as the Japanese dataset.

### 2.3 Categorization

We defined the categories and tags for answers for ease in analyzing their quality. We first defined the categories according to the features of our e-learning system. Then, we defined tags to explicitly write semantic meanings of answers; for example, ways of adding other teachers to the class and ways of removing old teachers. From this process, we finally defined 11 categories of answers: **Documents (C1)**, **Assignments (C2)**, **Test/Questionnaire (C3)**, **Contents (C4)**, **Uploading (C5)**, **Registration (C6)**, **Aggregation (C7)**, **Login (C8)**, **Contact (C9)**, **Students (C10)**, and **Basic Usage (C11)**. Table 3, Table 4 show statistics of the whole published dataset and numbers of questions and answers for each category, respectively.

### 2.4 Statistical Analysis

For quality control, we performed statistical analyses to evaluate how well the dataset is organized to train chatbot models. Note that the tags for answers can be seen as categories for questions as each answer has several questions. We use the following measurements that are widely used to evaluate the qualities of datasets.

1. Calinski and Harabaz (CH). CH is one of the most popular measures of cluster quality [2]. Intuitively, if all the data in a cluster are close to each other, but the data in different clusters are not close, then we can say that the quality of the clusters is high. The formal definition is given as follows:

(1) $$CH\left(k\right)=\;\frac{\left(n-k\right)\;B\left(k\right)}{\left(k-1\right)\;W\left(k\right)}$$

where *B*(*k*) and *W*(*k*) are the sums of squares for the *k* clusters of the inner- and inter-clusters, respectively, and *n* is the number of clustered data. Thus, the higher the score, the better the quality of the cluster.

2. Mutual Information (MI). MI is a popular information-theoretic measure. This measures the dependence between two sets; in other words, how much information values of a given category generates about the other given category. Thus, the lower the score, the better the quality of our dataset. The formal definition of this measure is as follows:

$$MI\left(A,\;B\right)=\;\underset{b\in B}{\sum }\underset{a\in A}{\sum }P\left(a,\;b\right)\log\frac{P\left(a,\;b\right)}{P\left(a\right)\;P\left(b\right)}$$

where A and B be the categories, *P*(*a*) and *P*(*b*) be the marginal probabilities, and P(a, b) be a joint probability. To evaluate the published dataset, we use a variant of MI called Adjusted MI (AMI) [3] defined as follows:

$$AMI\left(A,\;B\right)=\;\frac{MI\left(A,\;B\right)-E(MI\left(A,\;B\right))}{max\left(H\left(A\right),\;H\left(B\right)\right)-E(MI\left(A,\;B\right))}$$

where E(MI(A,B)) is the expected MI between the given two categories, max is a function returning the largest value of the given ones, and H(A) is the entropy of the category A.

3. Jaccard Index (Jaccard). This method calculates similarity by the number of common unique data shared by two given sets. To reduce errors caused by too large/small sizes of the given sets, this measure normalizes the results by dividing the size of the intersection of the two given sets by the size of their union. The formal definition is given as follows:

$$Jaccard\left(S_{A},\;S_{B}\right)=\;\frac{\left|S_{A}\cap^{}S_{B}\right|}{\left|S_{A}\cup^{}S_{B}\right|}$$

where $S_{A}$ and $S_{B}$ be sets of the given two categories.

4. TF-IDF + Kullback–Leibler (KL) divergence. TF-IDF is one of the most popular methods to measure the importance of a word in a document. KL divergence is an entropy-based method for measuring the similarity between two probability distributions. Their formal definitions are:

$$TF-IDF\left(w,\;d\right)=\;tf_{w,\;\;d}\frac{\left|D\right|}{\left|\text{\{}d^{'}\in D\;\text{|}\;w_{i}\in W\left(d'\right)\}\right|}$$

(2) $$KL\left(A,\;B\right)=\;\underset{a\in A,\;b\in B}{\sum }a\;\log\frac{a}{b}\;$$

where $tf_{w,\;\;d}$ be a term frequency of w in a document d and W(d) be a set of words of d. After creating TF-IDF vectors for all elements of A and B, we apply Eq. 2 for all combinations of the vectors.

5. TF-IDF + Jensen–Shannon (JS) divergence. JS divergence is an extension of KL divergence to be symmetric form.

$$JS\left(A,\;B\right)=\;\frac{1}{2}KL\left(A,\;M\right)+\;\frac{1}{2}KL\left(M,\;B\right)$$

where M be $\frac{1}{2}\left(A+B\right)$.

We attached the scores of the five measurements to our database (See metadata in Table 4). We believe that these scores can be useful to compare our study performed in Ref. [1] with subsequent new research on chatbots to investigate differences in detail or to allow transfer or extension of the dataset to another one.

As our e-learning system extends Sakai [4] without removing major functions that are usually implemented in e-learning system or Learning Management System (LMS) like distributing materials used in class, managing assignment, grades, and communication between teachers and students, it is able to use the questions about the functions in the case that a new dataset is created for another e-learning system. However, answers should be changed as there are several unique implementations of the functions and management by human e.g., how to restrict setting new passwords to respect our university's policy, which email address users should send if they cannot resolve the confront difficulties, and so on. Note that the provided statistical results are performed on questions' texts; thus, changing answers' texts does not affect the results.
