# Supplementary material for: An FAQ dataset for E-learning system used on a Japanese University
Source: Data Brief. 2019 May 24;25:104001. doi: 10.1016/j.dib.2019.104001 (PMC6595272; doi:10.1016/j.dib.2019.104001)
Supplement: Multimedia component 1 [file mmc1.doc]

Conflict of Interest and Authorship Conformation Form

Please check the following as appropriate:

- All authors have participated in (a) conception and design, or analysis and interpretation of the data; (b) drafting the article or revising it critically for important intellectual content; and (c) approval of the final version.
- This manuscript has not been submitted to, nor is under review at, another journal or other publishing venue.
- The authors have no affiliation with any organization with a direct or indirect financial interest in the subject matter discussed in the manuscript
- The following authors have affiliations with organizations with direct or indirect financial interest in the subject matter discussed in the manuscript:

Author’s name Affiliation

Yasunobu Sumikawa Tokyo Metropolitan University

Masaaki Fujiyoshi Tokyo Metropolitan University

Hisashi Hatakeyama Tokyo Metropolitan University

Masahiro Nagai Tokyo Metropolitan University
